# Tension Pneumocephalus Caused by Ethmoidal Roof Fracture: Emergent Surgical Decompression

**DOI:** 10.3390/diagnostics13010092

**Published:** 2022-12-28

**Authors:** Hak Sung Kim, Jae Ho Kim, Dae Kyun Kim, Sang Woo Ha

**Affiliations:** Department of Neurosurgery, College of Medicine, Chosun University, Gwangju 61453, Republic of Korea

**Keywords:** pneumocephalus, ethmoid bone, craniotomy

## Abstract

Tension pneumocephalus is a neurosurgical emergency that occurs when air is trapped in the intracranial cavity, leading to brain compression and causing severe neurological symptoms such as decreases in motor function, sensory function, and consciousness. Most cases of pneumocephalus require conservative treatment; however, because of the possible fatal complications, rapid diagnosis and appropriate treatment are important. Here, we present the case of an 81-year-old male patient who had undergone head trauma three hours prior to being admitted to our emergency room (ER) because of mental cloudiness. The radiologic findings showed tension pneumocephalus caused by an ethmoidal roof fracture. Emergency reconstruction of the ethmoidal roof with craniotomy was performed to remove the intracranial air using normal saline irrigation. By sharing our experience with this case, we hope to provide an option for the treatment of such cases.

**Figure 1:**
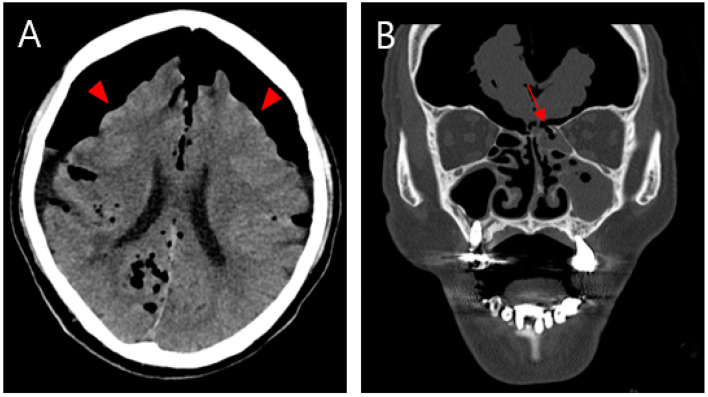
(**A**) Non-contrasting brain CT image showing pneumocephalus between the tips of the frontal lobes and the interhemispheric fissure, causing brain compression and demonstrating the Mount Fuji sign (arrowhead). (**B**) CT image of the paranasal sinus shows a fracture of the left ethmoidal roof (arrow). An 81-year-old male patient was admitted to our emergency room (ER) with mental cloudiness. Three hours prior to admission, he had slipped and tripped over a branch, injuring his nose. His initial Glasgow Coma Scale (GCS) was 11 (eye, 2; verbal, 3; motor, 6) and he had dyspnea upon arrival at the ER. Despite the high-pressure oxygen mask therapy, the continuous increase in oxygen demand required intubation and ventilator support. A physical examination revealed swelling, redness and bruising around his nose and a large amount of bloody rhinorrhea. A neurological examination revealed no specific findings other than decreased consciousness with mildly increased markers of infection in the laboratory examination. Computed tomography (CT) of the brain showed extensive pneumocephalus with brain compression, described as the Mount Fuji sign, and a CT of the paranasal sinus revealed a fracture of the left ethmoidal roof (Figure 1). A chest CT for evaluating lung problems revealed pneumonia in both lungs. With the diagnosis of tension pneumocephalus induced by an ethmoidal roof fracture, we performed combined surgery with an otolaryngologist. Through an endoscopic approach, cerebrospinal fluid (CSF) leakage was observed at the horizontal (cribriform) plate of the ethmoid area with a bone defect (6 mm). Closure of the dural and bone defects was performed using a bone graft, a mucosal flap, and fibrin sealant (Evicel) (Figure 2). After reconstruction of the ethmoidal roof, Burr hole trephination in the right frontal area was carried out with normal saline irrigation (Figure 3A). However, craniotomy was performed to remove the persistent intracranial air that caused brain compression because of aggravation of the neurological symptoms and no improvement in brain imaging. According to the post-operative CT, the pressure on the brain was reduced by removing the air, which thus improved the pneumocephalus (Figure 3B). After admission to the intensive care unit (ICU), postoperative conservative treatment was applied, including bed rest, supplemental oxygen therapy, and empirical antibiotic treatment for meningitis; central nervous system (CNS) infection was suggested by an analysis of the CSF. The patient gradually improved over a period of three weeks of hospitalization, as did the pneumonia, after which he breathed well without oxygen supplementation. At the time of discharge, his GCS score was 15, which was an improvement compared with the first time. At the one-month follow-up visit, the patient had no neurologic symptoms, and there were no specific findings revealed by brain CT and nasal endoscopy (Figure 4). Pneumocephalus is defined as the presence of air or gas in the intracranial cavity. The most common cause is head trauma; other reasons include neurosurgeries and spontaneous occurrences caused by tumors in the base of the skull, other tumors, infections, and barotrauma [1,2,3,4]. There is still much controversy about the mechanism; however, the most common theory can be explained by Horowitz’s “inverted soda bottle effect”. As a result of physiological drainage during straining, coughing, sneezing, the Valsalva maneuver, or iatrogenic lumbar drainage, excess loss of CSF leads to negative pressure in the cranial cavity [5,6]. Likewise, our case can be explained as massive CSF leakage due to an ethmoidal roof fracture, which trapped air through intracranial pressure. Pneumocephalus can be classified into two types: simple and tension. Patients with simple pneumocephalus are mostly asymptomatic. Sometimes, it can cause headaches, confusion, nausea, vomiting, and focal neurological symptoms. In most cases, conservative treatment, such as supplemental oxygen therapy [7], is required. However, in cases of tension pneumocephalus, which can cause brain compression and midline shifting to severe neurological symptoms, active management such as surgical decompression is required, and treating the primary cause is essential [8]. In this case, even though ethmoidal roof repair and burr-hole trephination were performed in light of the diagnosis of tension pneumocephalus with an ethmoidal roof fracture, craniotomy was carried out because of the persistent neurological symptoms after the operation. The patient showed an improvement in his symptoms after the operation. As tension pneumocephalus can cause severe neurological symptoms, rapid diagnosis and aggressive treatment should also be required by considering craniotomy.

**Figure 2:**
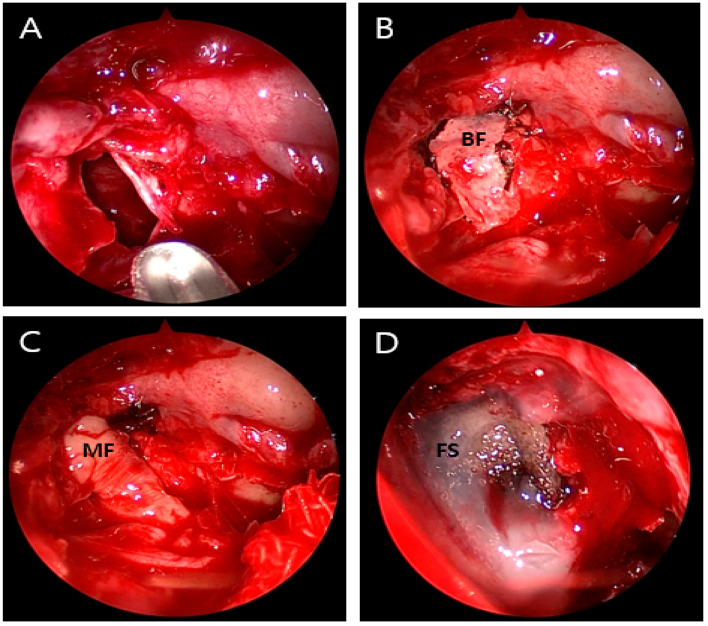
Intraoperative view images showing multi-layered closure of the dural and ethmoidal roof defect. (**A**) Bone defect (6 mm) on the horizontal (cribriform) plate of the ethmoid area. (**B**) Bone flap (indicated by ‘BF’). (**C**) Mucosal flap (MF). (**D**) Fibrin sealant (Evicel^®^) (FS).

**Figure 3:**
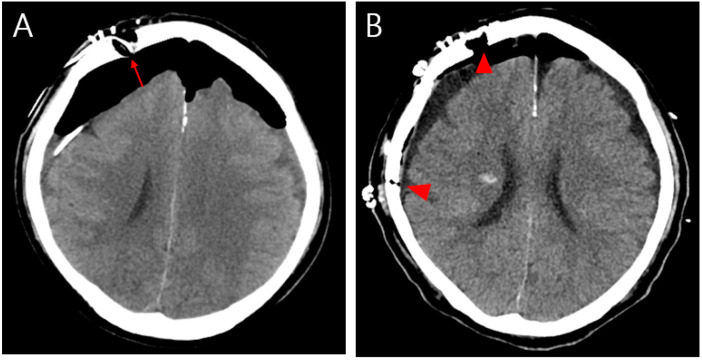
(**A**) Postoperative brain CT showing persistent intracranial air that compressed the brain parenchyma after burr hole trephination on the right frontal area (arrow). (**B**) After craniotomy, the CT image of the brain showed an improvement in pneumocephalus upon a reduction in the intracranial air (arrowhead).

**Figure 4:**
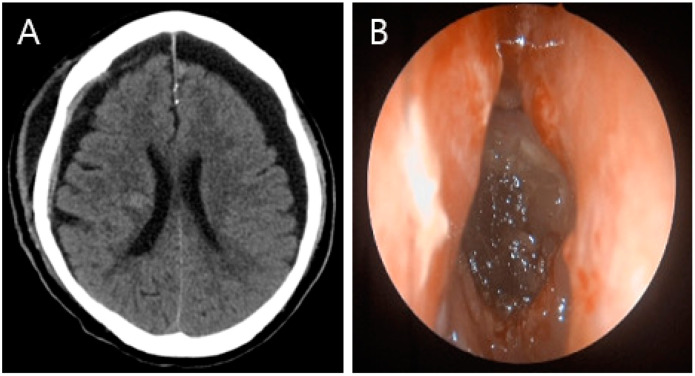
One-month follow-up. (**A**) Brain CT image, showing no specific findings except for bilateral hygroma. (**B**) Nasal endoscopy, showing that the defective area healed well without any specific findings.

## Data Availability

Not applicable.
